# Jupiter's X‐Ray and UV Dark Polar Region

**DOI:** 10.1029/2021GL097390

**Published:** 2022-06-07

**Authors:** W. R. Dunn, D. M. Weigt, D. Grodent, Z. H. Yao, D. May, K. Feigelman, B. Sipos, D. Fleming, S. McEntee, B. Bonfond, G. R. Gladstone, R. E. Johnson, C. M. Jackman, R. L. Guo, G. Branduardi‐Raymont, A. D. Wibisono, R. P. Kraft, J. D. Nichols, L. C. Ray

**Affiliations:** ^1^ Mullard Space Science Laboratory University College London Dorking UK; ^2^ The Centre for Planetary Science at UCL/Birkbeck London UK; ^3^ School of Physics and Astronomy University of Southampton Southampton UK; ^4^ School of Physics Trinity College Dublin Dublin Ireland; ^5^ Laboratoire de Physique Atmosphérique et Planétaire STAR Institute Université de Liège Liège Belgium; ^6^ Key Laboratory of Earth and Planetary Physics Institute of Geology and Geophysics Chinese Academy of Sciences Beijing China; ^7^ College of Earth and Planetary Sciences University of Chinese Academy of Sciences Beijing China; ^8^ Department of Science St. Gilgen International School St. Gilgen Austria; ^9^ School of Cosmic Physics DIAS Dunsink Observatory Dublin Institute for Advanced Studies Dublin Ireland; ^10^ Division of Space Science and Engineering Southwest Research Institute San Antonio TX USA; ^11^ Department of Physics and Astronomy University of Texas at San Antonio San Antonio TX USA; ^12^ Department of Physics Aberystwyth University Ceredigion UK; ^13^ Laboratory of Optical Astronomy and Solar‐Terrestrial Environment School of Space Science and Physics Institute of Space Sciences Shandong University Weihai China; ^14^ Harvard‐Smithsonian Center for Astrophysics Smithsonian Astrophysical Observatory Cambridge MA USA; ^15^ Department of Physics and Astronomy University of Leicester Leicester UK; ^16^ Department of Physics Lancaster University Lancaster UK

**Keywords:** Jupiter, Hubble Space Telescope, magnetosphere, dark polar region, aurora, Chandra X‐ray Observatory

## Abstract

We present 14 simultaneous Chandra X‐ray Observatory (CXO)‐Hubble Space Telescope (HST) observations of Jupiter's Northern X‐ray and ultraviolet (UV) aurorae from 2016 to 2019. Despite the variety of dynamic UV and X‐ray auroral structures, one region is conspicuous by its persistent absence of emission: the dark polar region (DPR). Previous HST observations have shown that very little UV emission is produced by the DPR. We find that the DPR also produces very few X‐ray photons. For all 14 observations, the low level of X‐ray emission from the DPR is consistent (within 2‐standard deviations) with scattered solar emission and/or photons spread by Chandra's Point Spread Function from known X‐ray‐bright regions. We therefore conclude that for these 14 observations the DPR produced no statistically significant detectable X‐ray signature.

## Key Points

- We compare 14 simultaneous Chandra X‐ray Observatory and Hubble Space Telescope UV observations of Jupiter's Northern Aurorae
- We detect no statistically significant X‐ray emission from the UV dark polar region (DPR)
- The DPR expands and the X‐ray and UV emission on the swirl region boundary shifts when the aurora show solar wind compression morphology

## 1 Introduction

Jupiter generates bright and dynamic ultraviolet (UV) and X‐ray aurorae at its poles. Jupiter's UV aurora are typically grouped into four components: (a) footprints of the Galilean satellites (e.g., Bhattacharyya et al., 2018; Bonfond et al., 2009, 2013; Hue et al., 2019; Szalay et al., 2018), (b) the main emission (e.g., Grodent et al., 2008), (c) emissions between the satellite footprints and main emission (e.g., Kimura et al., 2015; Mauk et al., 2002; Wibisono et al., 2021; Yao et al., 2020), and (d) emissions poleward of the main emissions (e.g., Grodent, 2015, and references therein). The main focus of this study is those emissions poleward of the main emission. To place these in context, we briefly introduce the main emission first.

**Figure 1:**
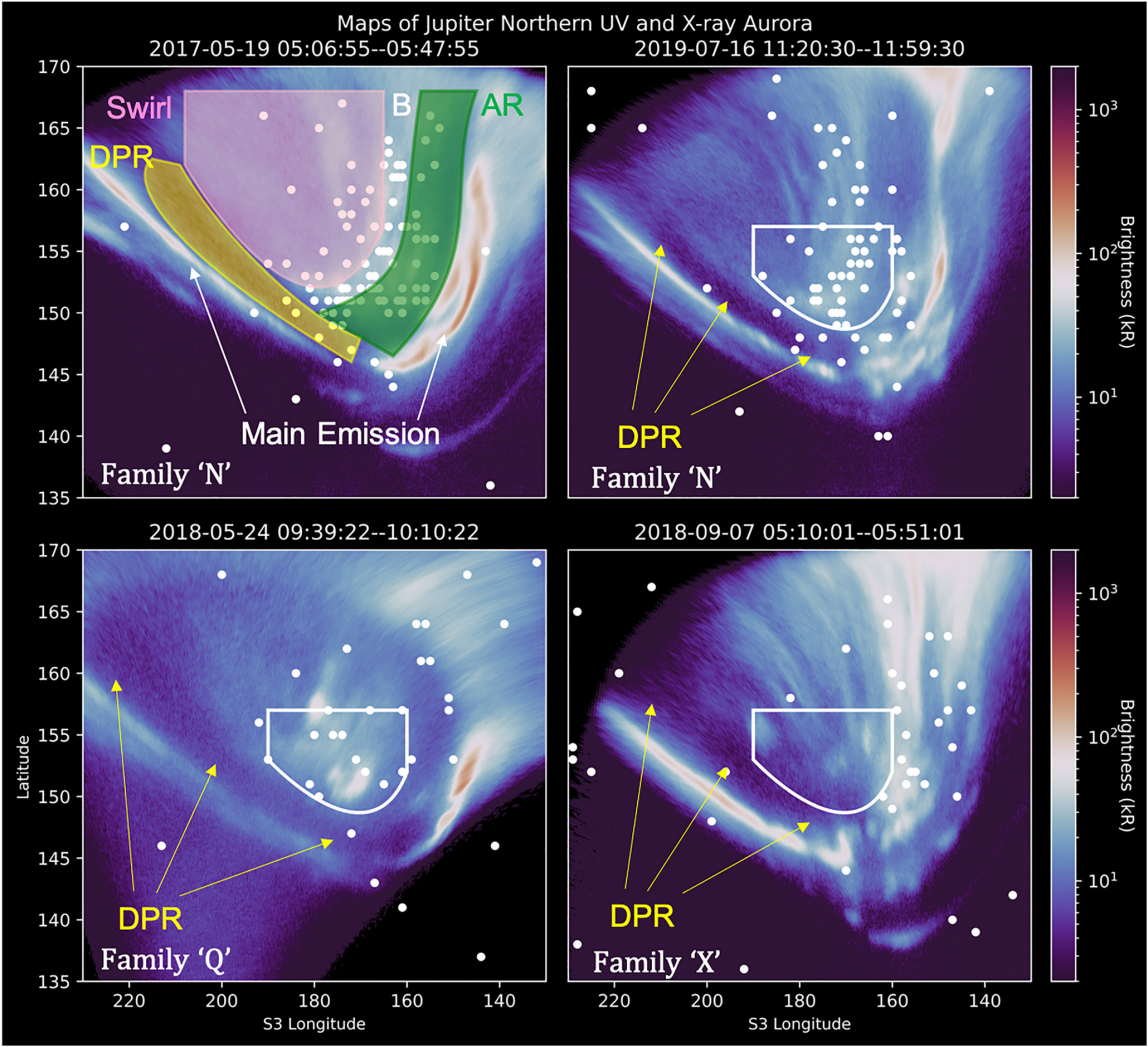
Overlaid simultaneous UV (blue‐white‐red color map) and X‐ray photon (white dots) longitude‐latitude maps of Jupiter's North Pole, from the Hubble Space Telescope (HST) and Chandra X‐ray Observatory High Resolution Camera (CXO‐HRC). Dates and times of the observations (UT) are at the top of each panel. Only UV and X‐ray emissions produced during this time window are shown. The top left panel highlights different aurora regions, as discussed in the introduction. The main emission is labeled by white arrows, the dark polar region (DPR) is shown in yellow, the Swirl region is shown in pink and the Active Regions (sometimes split into a noon and dusk active region) are shown in Green. The boundary between the active region and swirl region (here labeled with a white “B”) sometimes includes an arc of UV emission, as is the case for the two different observations shown in the top two panels here. The other panels highlight three different UV aurora families, as indicated by the white label in the lower left corner of each. The white shape overlaid onto each map is consistent across each, and highlights the changing spatial distribution of X‐rays for each. For each panel, the location and extent of the DPR are indicated with yellow arrows, showcasing its changing extent from observation‐to‐observation. We note that due to the viewing geometry from Earth and the tilt of Jupiter's pole, visibility of the most poleward latitudes is limited. For consistency, we cutoff the latitude on the maps at 170°.

The most easily recognized feature of Jupiter's UV aurora is the main auroral emissions (labeled in Figure 1). These form a quasi‐continuous arc around the magnetic pole and are generally associated with a large‐scale current system that connects to the middle magnetosphere (Cowley & Bunce, 2001; Grodent, Clarke, Kim, et al., 2003; Hill, 2001; Nichols et al., 2009; Yao et al., 2019). Recent results from the Juno spacecraft show that this region contains both upward and downward currents (Kotsiaros et al., 2019; Mauk et al., 2020). The emissions in this region are produced by monodirectional electrons accelerated by both electrostatic potentials and broadband processes, with the brightest aurorae often produced by broadband processes (Mauk et al., 2017a, 2017b, 2020).

Poleward of this main emission there are several different auroral structures that include: the swirl region, the active regions, and the dark polar region (DPR; each indicated in Figure 1). The swirl region is the highest magnetic latitude region and occurs closest to the magnetic pole (Grodent, Clarke, Waite, et al., 2003; Masters et al., 2021; Nichols et al., 2017; Stallard et al., 2016). At magnetic latitudes below the swirl region but above the main emission, there are the active regions (e.g., Nichols et al., 2009) and DPR, which are sometimes grouped together as the “polar collar” (Greathouse et al., 2021) and encircle the swirl region. The active region occurs in the noon to dusk sectors and is sometimes separated as the “noon active region” and “dusk active region” due to the different behaviors of each (e.g., Nichols et al., 2009). Between the main emission and the swirl region, predominantly in the dawn sector, lies a region mostly devoid of UV emission: the DPR (e.g., Johnson et al., 2017; Swithenbank‐Harris et al., 2019).

The behaviors of these regions differ. While the active regions produce a variety of bright and impulsive flares (Bonfond et al., 2016; Waite et al., 2001), the swirl region produces slightly dimmer sporadic, short‐lived aurorae (Bunce et al., 2004; Grodent, Clarke, Waite, et al., 2003; Masters et al., 2021; Nichols et al., 2017; Stallard et al., 2016). Over long integration times, H_3_^+^ observations and UV observations sometimes show the presence of an arc along the boundary between the active region and swirl region (Johnson et al., 2017, 2018; Stallard et al., 2016). The Juno UVS instrument shows that while the swirl region is controlled by ionospheric local time (dictated by the direction of sunlight), the “polar collar” is controlled by magnetospheric local time (Greathouse et al., 2021). The emissions from the swirl region also appear to be connected with regions deeper in the atmosphere, with more absorption from methane, than the active region (Greathouse et al., 2021).

Jupiter's UV aurora can be grouped into six families of behavior. Grodent et al. (2018) defined these as Q, N, U, i, I, and X. Examples of families N, Q, and X are shown in Figure 1. “Q” has a low (quiet) overall auroral power and an expanded main emission. “N” has a narrow main emission and an average power, while U is the “Unsettled” intermediate between “Q” and “N.” Family “i” has injection signatures but a continuous main emission, while “I” has strong injection signatures with a disrupted dawnside emission and a “corner‐type” structure in the injection signatures. Finally, “X” is indicative of external perturbations, with strong dawnside main emissions, strong arcs in the duskside, and a contracted main emission. This morphology is most commonly observed during solar wind compressions. For further details see Grodent et al. (2018).

Previous work exploring Jupiter's X‐ray and UV auroral spatial connections was limited to a single simultaneous Chandra X‐ray Observatory (CXO) and Hubble Space Telescope (HST) observation on 23 February 2003 (Branduardi‐Raymont et al., 2008; Elsner et al., 2005). This joint observation revealed two key connections between the wavebands: (a) X‐ray bremsstrahlung emissions from precipitating electrons coincide with the UV main emission (Branduardi‐Raymont et al., 2008) and (b) X‐ray emissions from precipitating ions coincide with UV flares in the active region (Elsner et al., 2005). A recent spectral and timing study of the X‐ray and UV aurora (Wibisono et al., 2021) suggested that auroral injection events and dawn storms (Yao et al., 2020) may also coincide with an X‐ray bremsstrahlung component. In analyzing the X‐ray, UV, and radio observations from 2007, there was found to be a difference between pulsed/flared X‐ray emissions and a steady “flickering” X‐ray aurorae, with both distinct aurorae produced by precipitating ions (Dunn, Gray, et al., 2020). Recent work has shown that the X‐ray flares are correlated with compressional waves and electromagnetic ion cyclotron (EMIC) waves in the outer magnetosphere (Yao et al., 2021), suggesting that the periodic EMIC waves cause the periodic ion precipitation that produces the X‐ray flares. Dunn, Branduardi‐Raymont, et al. (2020) and Dunn, Gray, et al. (2020) found that for observations when the X‐ray aurora were dominated by pulsed/flared X‐ray emission, the spectrum was better fit by atomic charge exchange spectral lines from Iogenic ions (Sulfur and oxygen). However, during the two observations when flickering X‐ray auroral emission was also present, comparable or slightly better fits were achieved by including solar wind ions in the spectral models (using the ion abundances from Von Steiger et al. [2000]). For pulsed aurorae, Iogenic ion precipitation X‐ray spectrum achieved model fits with $\chi^{2}=0.85-0.9$, where solar wind ion models only produced fits of $\chi^{2}=$ 1.2. In contrast, for flickering aurorae intervals, models produced fits of $\chi^{2}=1.1-1.3$ for purely iogenic models, but $\chi^{2}=1-1.1$ for solar wind models. Potentially suggesting, changing ion populations or acceleration with changing auroral emission.

While these previous X‐ray and UV studies concentrated on the presence of auroral emissions, the absence of emissions can also be indicative of the underlying magnetospheric processes. Here, we leverage the necessary spatial resolution of the CXO to explore the prevalence of X‐ray emission in the DPR. Previous work has explored the possible source for this structure. IR observations analyzed by Johnson et al. (2017) showed that the DPR significantly lags behind corotation with the planet by 2.2 km s^−1^ and is approximately stationary relative to the magnetic pole. They found that better spatial resolution (or possibly a change in the Jovian aurora) enabled them to more clearly distinguish the DPR flows from the swirl region flows, previously thought to be the stagnant structure (Stallard et al., 2003). Building on the theoretical work of Cowley et al. (2003), Johnson et al. (2017) suggested that the stationary polar flows could be due to the region's connection to open flux convecting with the solar wind through a single‐cell Dungey cycle in the dawn sector. Delamere and Bagenal (2010) proposed an alternative that still connected the region to the solar wind but via viscous drag as the field lines open and closes along the dawn boundary. However, intermittent Kelvin Helmholtz reconnection along this boundary should potentially lead to auroral signatures, but the region appears to be devoid of such emissions (Johnson et al., 2017; Swithenbank‐Harris et al., 2019). Recent Jovian magnetosphere modeling suggests that Jupiter's open flux could be stretched into a crescent‐like structure poleward of the main emission and that the rotation of the system should produce a twisted‐bundle of closed flux in the dawn sector either of which may produce the DPR signature (Zhang et al., 2021). A final proposed explanation is that the region could be the downward current region, closing upward current systems flowing away from the planet (Swithenbank‐Harris et al., 2019).

An absence of UV emission suggests low fluxes of precipitating energetic electrons. In contrast, X‐ray auroral spectra consist of both continuum emissions from precipitating electrons and spectral lines from precipitating ions (Branduardi‐Raymont et al., 2004, 2007a; Cravens et al., 1995; Dunn, Branduardi‐Raymont, et al., 2020; Elsner et al., 2005; Houston et al., 2020; Kharchenko et al., 2008; Wibisono et al., 2020). The persistent detection of X‐ray aurora ion spectral lines led to the suggestion that Jupiter's X‐ray aurorae may trace Jupiter's downward current regions (Cravens et al., 2003). If this is the case, then the presence of soft X‐ray signatures in the DPR would be consistent with the proposed downward current region. Alternatively, if the DPR represents Jupiter's cusp, then it may be that X‐ray emissions from solar wind charge exchange (SWCX) could be present at the boundary (e.g., Dunn, Gray, et al., 2020). To explore these possibilities, we compare the distribution of X‐ray emissions with the location of the DPR in a new set of simultaneous UV‐X‐ray aurora observations.

## 2 Observations

The HST and CXO observations presented here were part of the observing programs for Juno's approach to Jupiter and for Juno's orbits 3–22. The HST observations were undertaken with the Space Telescope Imaging Spectrograph (STIS) UV camera, which offers a ∼25 × 25'' (arc second) field of view with a 0.024''^2^ plate‐scale. The CXO observations were taken with the High Resolution Camera (HRC). CXO‐HRC offers a much wider field of view (30 × 30'), enabling observations of Jupiter and its moons (Elsner et al., 2002; Nulsen et al., 2020) simultaneously, but with lower spatial resolution, with a pixel size of 0.13'' and a Full Width Half Maximum (FWHM) through the instrument optics of 0.4'' Consequently, for the purposes of this study, we consider the limitations on the CXO‐HRC spatial resolution as the dominant uncertainty on the co‐location of UV and X‐ray events, and only include this within the mapping. The 1‐sigma (0.4'') uncertainty in the sky‐projected location of an X‐ray is projected into System III coordinates to produce a longitude‐latitude uncertainty for each photon, which we label as the “photon spatial uncertainty.” Both HST‐STIS and CXO‐HRC can operate in time‐tagged mode, storing the time at which each event arrived at the detector to enable the building of maps and videos of emission. Chandra's time resolution is sufficiently short (16 μs) such that we do not include this within the mapped uncertainties. For further instrument setup for HST see Nichols et al. (2017) and Grodent et al. (2018) and for CXO see Weigt et al. (2020). Finally, we note that for the X‐ray photon energies associated with Jupiter's aurorae, CXO‐HRC has very limited energy resolution such that it is not possible to identify the energy of individual X‐ray photons or produce spectra to distinguish spectral lines from precipitating ions undergoing charge exchange from continuum emissions associated with bremsstrahlung from precipitating electrons.

These HST and CXO campaigns offer a new set of simultaneous UV and X‐ray observations of the solar system's most powerful aurorae. The work presented here represents the first in a series of studies that leverage this simultaneous data set to explore spatial and temporal connections between the UV and X‐ray auroral emissions. Here, we focus only on the DPR. This work was undertaken with the Orbyts program, which partners scientists with schools to support school students' involvement in research (Chubb, Joseph, et al., 2018; Chubb, Naumenko, et al., 2018; Darby‐Lewis et al., 2019; Edwards et al., 2020; Francis et al., 2020; French et al., 2020; Grafton‐Waters et al., 2021; Holdship et al., 2019; McKemmish et al., 2017, 2018; Wibisono et al., 2020).

Table 1 lists every simultaneous observation of Jupiter's Northern aurora, with the accompanying UV family as defined in Grodent et al. (2018). X‐ray photons were only included from times during which HST observed. Jupiter's Northern aurorae are best viewed for Central Meridian Longitude (CML) values close to 160°. We caution the interpretation of connections between the X‐ray and UV locations for the observations on 2016‐05‐24 T20:56‐21:40 and 2016‐06‐01 T18:08‐18:52, when the aurorae were close to the limb of the planet. This CML range combined with the small Jupiter disk size on the sky (diameter of 37–38'' compared with e.g., 45'' for 2018‐05‐24), due to the high Jupiter‐Earth distance, leads the System III uncertainty for a given auroral photon to cover the majority of the auroral zone. Given the limited number of available observations, we include these high spatial uncertainty observations in the table, with this note of caution.

**Table 1:** X‐Ray Counts From the DPR for the Time Intervals Shown in the First Column

CXO‐HST joint observation	CML /°	Jupiter‐Earth distance/AU	UV family	DPR photons SIII long	DPR photons SIII long <190°	Region X photons	Expected active and swirl region X‐rays detected in DPR	Mean solar scattered photons
YYYY‐MM‐DD T hh:mm‐hh:mm				>190°				
2016‐05‐24 T17:45‐18:29	93–119	5.18	Q	0	0	26	0.78 ± 0.87	0.36 ± 0.21
2016‐05‐24 T20:56‐21:40	208–235	5.18	U	1	2	23	0.55 ± 0.73	0.64 ± 0.69
2016‐06‐01 T14:57‐15:41	114–141	5.29	N	1	3	12	1.33 ± 1.1	1.37 ± 0.38
2016‐06‐01 T18:08‐18:52	230–256	5.29	Q/N	3	0	9	0.74 ± 0.83	0.93 ± 0.,74
2017‐02‐02 T16:58‐17:38	101–125	5.04	I	0	1	11	0.19 ± 0.43	0.31 ± 0.21
2017‐03‐27 T08:41‐09:21	226–250	4.48	I	0	0	7	0.11 ± 0.32	0.26 ± 0.4
2017‐05‐19 T05:06‐05:46	159–183	4.69	N	0	4	47	1.66 ± 1.26	0.53 ± 0.31
2017‐05‐19 T06:42‐07:22	217–241	4.69	N	0	0	24	0.93 ± 0.95	0.58 ± 0.66
2018‐04‐01 T10:38‐10:56	161–172	4.62	N	0	0	14	0.66 ± 0.79	0.28 ± 0.21
2018‐05‐24 T09:39‐10:09	191–209	4.43	Q	0	1	15	0.59 ± 0.75	0.88 ± 0.55
2018‐09‐07 T05:10‐05:50	137–161	5.75	X	1	0	1	∼0	0.76 ± 0.34
2019‐07‐15 T14:43‐15:21	119–142	4.44	N	1	2	17	2.11 ± 1.38	0.93 ± 0.42
2019‐07‐15 T16:16‐16:54	175–198	4.44	Q	1	2	14	0.67 ± 0.8	0.6 ± 0.45
2019‐07‐16 T11:20‐11:58	147–170	4.45	N	1	2	24	1.9 ± 1.3	0.49 ± 0.26

*Note*. Northern aurora X‐ray photons are only included from the time window in the first column. We distinguish the DPR photons with System III longitudes greater and less than 190°, as an indicator for events occurring close to the active and swirl regions. The penultimate columns show the number of photons from the active and swirl region that are expected to be detected in the whole DPR because of uncertainties in the photon spatial location (see e.g., Figures 3c and 3d) as mean ± standard deviation from 100,000 simulation runs. The final column shows the number of scattered solar photons that are expected to be detected in the DPR during the HST observation (see Figures 3e and 3f), as mean ± standard deviation from the scattered solar photon measurements. The scattered solar photons are measured from the complete Chandra observations, but the count‐rate is scaled to the HST observation duration.

**Figure 2:**
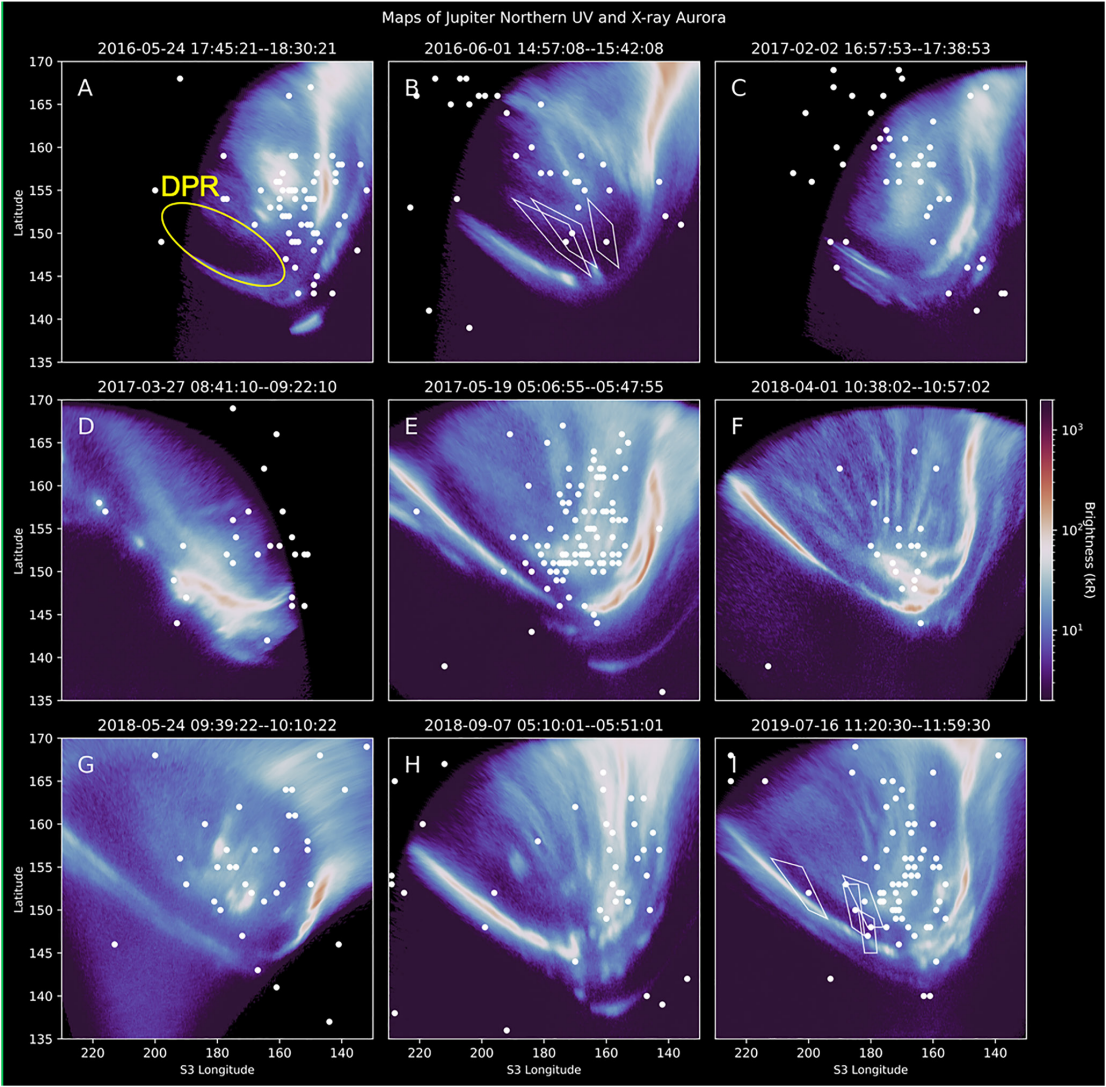
Overlaid simultaneous UV (blue‐white‐red color map) and X‐ray photon (white dots) longitude‐latitude maps of Jupiter's North Pole, from the Hubble Space Telescope (HST) and Chandra X‐ray Observatory High Resolution Camera (CXO‐HRC). Dates and times of the observations (UT) are at the top of each panel. Only UV and X‐ray emission produced during these times is shown. Panel (a) shows a yellow circle indicating the location of the dark polar region (DPR). Panels (b and i) show white quadrilaterals around example X‐ray photons. The vertices of a given quadrilateral are the extremes of the photon's possible projected longitude‐latitude location based on the projected 1‐sigma (0.4″) point spread function of CXO. See Table 1 for the CML range and Jupiter‐Earth distance for each observation.

**Figure 3:**
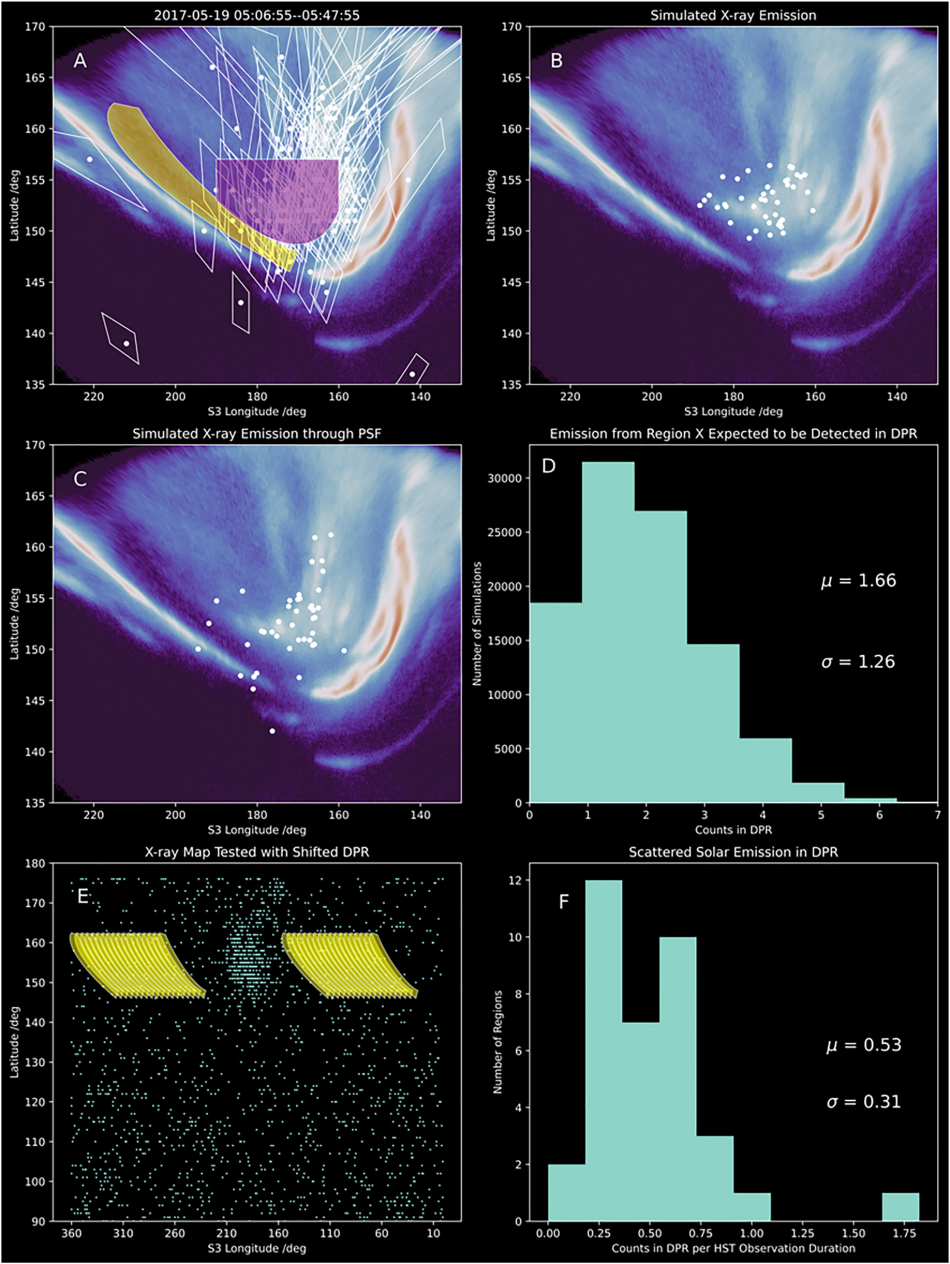
UV Observations and X‐ray Observations and Simulations from 19 May 2017. (a) shows an overlaid longitude‐latitude map of UV brightness (color‐map) and X‐ray photons (white dots) and their respective 1‐sigma uncertainty quadrilaterals (white lines around each dot) from Jupiter's Northern aurora on 19 May 2017 from 05:06 to 05:47. The yellow shaded region indicates the DPR at this time as defined from the UV observations, while the purple region shows Region X defined to combine X‐ray emissions observed from the active and swirl region, with 1‐sigma uncertainties that could enable instrument scattering of the photons into the DPR. (b) shows simulated X‐ray emission from within the purple region in (a), as outlined in the main text. (c) applies Chandra's point spread function to the emission in (b) to apply the spatial response of the instrument to such emission. (d) shows the resulting histogram from 100,000 simulations of the X‐ray emission and PSF, showing the number of photons initially produced in the purple region that would be detected in the DPR. (e) shows the Northern hemisphere X‐ray map from the entire 10‐hr observation of Jupiter by Chandra HRC‐I on 19 May 2017. Yellow regions show shifted DPR regions within which the number of photons was measured to quantify scattered solar X‐ray contributions within the DPR. (f) shows the resulting histogram of the number of X‐ray counts scaled for the ratio between the duration of the Chandra observation and the HST observation, to quantify how many scattered solar photons may contribute to the observed DPR emission within the simultaneous CXO‐HST time window. μ indicates the mean and σ the standard deviation for the distribution.

Figure 2 presents nine representative System III longitude‐latitude maps of Jupiter's Northern aurora from simultaneous HST‐CXO observations on nine different days from 2016 to 2019 (see Table 1 for observation date times). These show the mean UV brightness of the aurora (color bar) and all X‐ray photons (white dots) detected by the CXO during the HST observation window. Figure 2 and Table 1 show that for each observation there are between 0 and 4 X‐ray photons co‐located with the DPR. White quadrilaterals are shown plotted for a few example photons in Figures 2b and 2i. The vertices of a given quadrilateral are the extremes of the photon's projected longitude‐latitude location based on the 1−σ PSF of the CXO‐HRC. To plot these, we shifted the sky‐projected photon location by ±1−σ (0.4'') in the *x*‐direction and the *y*‐direction in the sky *x*‐*y* plane, then re‐projected the four resulting locations into System III coordinates. These “spatial uncertainty quadrilaterals” are only shown for a limited selection of photons to ensure that excess quadrilaterals do not inhibit the interpretation of the maps. Figure 3a shows an example with quadrilaterals plotted for all X‐ray photons on 2017‐05‐19 T05:06‐05:46.

## 3 Numerical Tests of Source Locations for DPR Photons

Figure 2 and Table 1 show that a low number of X‐ray photons are detected in the DPR for all observations in the catalog. Further, these photons are not uniformly detected across the DPR, but instead the majority are detected at system III longitudes of less than 190° and within spatial uncertainties of nearby bright UV and X‐ray auroral structures.

While spurious X‐ray events from the Chandra HRC are uncommon, because the instrumental background is low, it is important to consider two known non‐DPR sources that may contribute to the DPR photons: (a) solar photons scattered in Jupiter's upper atmosphere, which are present across the Jovian disk (Bhardwaj et al., 2005; Branduardi‐Raymont et al., 2007b; Cravens et al., 2006; Dunn et al., 2016; Maurellis et al., 2000) and (b) photons that may be from other bright auroral emissions, but due to the photon's spatial uncertainty may be detected in the DPR. We consider each possibility in turn.

For the first potential source, to sample the number of scattered solar photons that might be expected in the DPR, we took a region the size and shape of the observed DPR in the respective HST observation and shifted it in System III longitude to 30–100 new non‐auroral longitudes on the map. Figure 3e shows an example map of Jupiter's Northern hemisphere emission, with shifted DPR regions shown in yellow (both at smaller and larger longitudes than the auroral region shown in Figure 3a). Figure 3f shows an example resulting histogram of the counts detected in that region scaled to the HST observation duration. The final column of Table 1 shows the mean ± standard deviation of this expected scattered solar photon count distribution for each observation, with the mean ranging from 0.3 to 1.4 counts depending on the size of the DPR at this time, the distance to Jupiter, and the solar activity.

For the second potential source, we sought to test whether those photons coincident with the DPR could actually be swirl/active region emission spread into the DPR by the photon's spatial uncertainty. To do this, for each observation we created models that simulated the X‐ray emission from non‐DPR auroral emissions and tested to what extent the CXO PSF would move this emission so that it was detected in the DPR. To do this we:

1. **Defined a region of choice:** For each observation, we defined a region that we labeled “Region X.” This region was centered on the clustering of X‐ray photons detected in the UV active and swirl region, but did not include the DPR. The size of the region extended to include any photons in the active and swirl regions for which the spatial uncertainties could have led them to instead be detected in the DPR. This region is similar in location to the “X‐ray hot spot” region reported previously (Dunn et al., 2016, 2017; Gladstone et al., 2002; Kimura et al., 2016; Weigt et al., 2020), but the photons included in this region are only those for which the spatial uncertainties meant that they could have been detected in the DPR. Figure 3a shows Region X (in purple) and the DPR (in yellow) for the 2017‐05‐19 T05:06‐05:46 observation, and illustrates the size of the spatial uncertainties for each photon to show the limits on the size of region X. For all but one observation, the center of the clustering of X‐rays was within 5° longitude‐latitude of 172° longitude and 155° latitude. Figure 1 shows the exception to this was the observation on 2018‐09‐07 (the only observation in UV family X), when the X‐rays were not clustered around the average Region X location (the white shape), and their spatial uncertainties did not reach the DPR. Having defined this region for each observation in turn, we counted the number of X‐ray photons within Region X and the DPR. These are recorded in Table 1.
2. **Simulated the X‐ray emission from Region X:** Each simulation run generated a number of photons equal to the total observed counts from Region X and the DPR for that observation. To determine the source latitude‐longitude of each simulated X‐ray we assumed that the X‐ray emissions in Region X preferentially occur closer to the bright UV emission (following Elsner et al. [2005] and the observations here). To do this, we took the UV brightness at each 0.1° longitude‐latitude step in Region X and divided this by the sum of the UV brightness in Region X to produce a probability for each longitude‐latitude. Each X‐ray's source location was chosen randomly using the probabilities from each longitude‐latitude in Region X. Figure 3b shows an example simulated emission from Region X (purple region) shown in Figure 3a. We note that we only simulated emission from inside Region X since we are seeking to test whether, when the spatial uncertainties of the instrument are accounted for, emission from this region can explain the observed X‐rays in the DPR. In the main text, we chose to use the UV brightness as our probability map because X‐ray emissions are highly variable from observation to observation (e.g., Dunn et al., 2016; Dunn, Gray, et al., 2020; Jackman et al., 2018; Weigt et al., 2021). In Supporting Information S1, we show results from simulating the X‐ray source regions using a uniform probability across Region X and note that the error bars on the mean from the uniformly distributed X‐ray photons overlap those from the UV‐distributed X‐ray photons.
3. **Applied the CXO PSF to the simulated emission:** Having simulated the expected X‐ray photon emission from Region X, we shifted the location of each simulated photon from the location where it was emitted in region X (Figure 3b) to the location where it would be detected after the instrument PSF was applied (Figure 3c). The direction of the latitude‐longitude shift was chosen at random. The extent of the shift was determined by a Gaussian probability distribution with a standard deviation given by the instrument PSF projected into that latitude‐longitude direction for a random time within the simultaneous HST‐CXO observing window.
4. **Counted the resulting number of photons in the DPR:** We simulated each observation 100,000 times, each time counting the number of X‐rays that were detected in the DPR from emission that originated in Region X.

The resulting mean ± standard deviation from 100,000 simulations for each observation is shown in the penultimate columns of Table 1. For all observations, the X‐ray photon counts in the DPR are within 2‐standard deviations of the scattered solar photons plus PSF‐scattered Region X photons. This suggests that there is not a statistically significant detection of X‐rays from the DPR for these 14 observations.

## 4 Discussion

In the introduction, we outlined three potential sources for the DPR: a downward current region (Swithenbank‐Harris et al., 2019), a Dungey‐like single‐cell open field and return flow (Cowley et al., 2003) or Kelvin Helmholtz instabilities in viscous interactions on the dawn flank (Delamere and Bagenal et al., 2010). Two further possibilities are that sufficient potential drops are not present in this location or that the magnetospheric local time sector connected with the DPR does not possess the broadband processes (e.g., wave‐particle interactions) that are present in the other regions (e.g., Mauk et al., 2017a, 2017b, 2020; Yao et al., 2021). Does the apparent lack of X‐ray emission from the DPR enable us to better constrain these possible suggestions?

Turning first to the possibility that the DPR is a downward current region: The ion precipitation that dominates Jupiter's X‐ray aurorae has long been considered to be indicative of downward currents (e.g., Cravens et al., 2003). The lack of X‐ray emission in the DPR in contrast with the active region or the boundary of the swirl, suggests that downward currents, potential drops, and/or wave‐particle interactions in the DPR must be significantly exceeded by those in the other polar aurorae, and/or the DPR downward currents are carried entirely by upward flowing electrons. Electron fluxes into the dark region were found to be much less intense than those flowing into the active region (Gérard et al., 2019). Ion fluxes and MV potential drops close to the regions associated with X‐ray auroral emissions have begun to be explored by the Juno spacecraft (Clark et al., 2020).

For the single‐cell Dungey model (Cowley et al., 2003), the stagnant flows in the DPR imply that the DPR would be the open field region, with the motion governed by the flow of the solar wind (Johnson et al., 2017). If the DPR is open to the solar wind, it seems that there is not sufficient solar wind ion precipitation to produce an observable solar wind charge exchange X‐ray signature. However, the initial solar wind precipitation would happen close to the boundary. The subsequent open field lines would be sparsely populated with particles after the initial precipitation, so that for open field lines the low detection of X‐rays and consequent low levels of precipitating particles may be consistent with this proposed source. Zhang et al. (2021) used MHD models to explore what auroral signatures a Jupiter‐like magnetosphere might produce. They found that inside of the main auroral emission a crescent‐like arc of open field lines would be present. This is similar to the DPR observed throughout these observations.

Kelvin Helmholtz instabilities may inject solar wind ions on the dawn flank (Delamere and Bagenal et al., 2010), but, as discussed above, any SWCX emission in the DPR is below the Chandra detection threshold for these observations. However, Dunn, Gray, et al. (2020) noted that solar wind ions are present in the spectra during low solar wind ram pressure. At these times, the magnetosphere would be more expanded and compressible, potentially favoring the entrance of a solar wind ion population through viscous processes.

Finally, we discuss the 2018‐09‐07 observation (shown in Figure 1) which may offer further clues. If this observation is indeed accompanied by a solar wind compression, as implied by the auroral morphology (Dunn, Gray, et al., 2020; Grodent et al., 2018; Nichols et al., 2009, 2017), then the expansion of the DPR at this time could be consistent with an expansion of the open field region. Although for Saturn, a solar wind compression can in fact close flux by triggering tail reconnection (e.g., Badman et al., 2016).

For all other auroral families, Region X required *a* <5° shift in latitude‐longitude to overlap with the densest concentration of X‐ray emission. However, for 2018‐09‐07 the X‐ray emission almost entirely shifted to the dusk arc so that no X‐ray photons were detected in the average Region X from the other 13 observations (white shape in Figure 1). The X‐ray emission along the bright boundary of the swirl region (e.g., Johnson et al., 2017; Stallard et al., 2016) also either shifted or vanished. One proposed source of X‐ray emissions is that they are produced by processes along the magnetopause (e.g., reconnection, Kelvin Helmholtz instabilities, and/or Chapman‐Ferraro currents) (Bunce et al., 2004, Dunn et al., 2016, 2017; Kimura et al., 2016; Weigt et al., 2020, 2021). A shifting of the swirl boundary may fit with a theoretical interpretation that involves a shifting of the ionospheric mapping of the magnetopause to lower latitudes caused by a solar wind compression. Alternatively, given that compressional waves and EMIC waves have been observed in many intervals to be strongly correlated with Jupiter's X‐ray flares (Yao et al., 2021), the shift of the boundary may indicate a shift in the location of outer magnetosphere processes.

## 5 Conclusion

We examined 14 simultaneous CXO and HST observations of Jupiter's Northern UV and X‐ray aurora, testing for evidence of auroral X‐ray photons produced in the DPR to differentiate between proposed explanations for the region. The very low levels of X‐ray photon counts detected in the region are consistent with the expected emission from scattered solar photons in the atmosphere and X‐rays shifted by the instrument point spread function from the nearby active region and boundary of the swirl region. This suggests that for these 14 observations the DPR did not produce any X‐ray emission that was detectable by CXO. In turn, this implies low levels of precipitation by energetic magnetospheric ions and solar wind ions in the DPR. The observations presented here are consistent with the DPR being either Jupiter's open field line region, or require the magnetospheric local time sector connected with the region to have different potential drops or to be absent of the strong downward currents or wave‐particle interactions associated with the rest of Jupiter's polar X‐ray aurora.

## Supporting information

Supporting Information S1

## Data Availability

All data presented in this work are publicly available through the Chandra archive (https://cda.harvard.edu/chaser/) and the Hubble Space Telescope data available from the MAST Archive with the approach campaign here: https://archive.stsci.edu/doi/resolve/resolve.html?doi=10.17909/t9-1271-7f52 and observations synchronized with the Juno orbits of Jupiter here: https://archive.stsci.edu/proposal_search.php?mission=hst&id=14634.
