# Supplementary material for: Jupiter's X‐Ray and UV Dark Polar Region
Source: Geophys Res Lett. 2022 Jun 7;49(11):e2021GL097390. doi: 10.1029/2021GL097390 (PMC9287093; doi:10.1029/2021GL097390)
Supplement: Supplementary file 1 — Supporting Information S1 [file GRL-49-0-s001.pdf]

AGU PUBLICATIONS

Geophysical Research Letters

Supporting Information for

Jupiter's X-ray and UV Dark Polar Region

W. R. Dunn[1,2], D. M. Weigt[3,4], D. Grodent[5], Z. H. Yao[6, 7], D. May[8], K. Feigelman[8], B. Sipos[8], D. Fleming[8], S. McEntee[4,9], B. Bonfond[5], G. R. Gladstone[10,11], R. E. Johnson[12], C. M. Jackman[9], R. L. Guo[13], G. Branduardi-Raymont[1,2], A. D. Wibisono[1,2], R. P. Kraft[14], J. D. Nichols[15], L. C. Ray[16]

[1]Mullard Space Science Laboratory, University College London, Dorking, UK.

[2]The Centre for Planetary Science at UCL/Birkbeck, Gower Street, London WC1E 6BT, UK.

[3]School of Physics and Astronomy, University of Southampton, Southampton, SO17 1BJ, UK
[4]School of Physics, Trinity College Dublin, Dublin, Ireland

[5]Laboratoire de Physique Atmosphérique et Planétaire, STAR institute, Université de Liège, Liège, Belgium.

[6]Key Laboratory of Earth and Planetary Physics, Institute of Geology and Geophysics, Chinese Academy of Sciences, Beijing, China.

[7]College of Earth and Planetary Sciences, University of Chinese Academy of Sciences, Beijing, China.

[8]Department of Science, St. Gilgen International School, St. Gilgen, Austria
[9] School of Cosmic Physics, DIAS Dunsink Observatory, Dublin Institute for Advanced Studies, Dublin 15, Dublin, Ireland

[10]Space Science and Engineering Division, Southwest Research Institute, San Antonio, TX, USA

[11]Department of Physics and Astronomy, University of Texas at San Antonio, San Antonio, TX, USA
[12]Department of Physics, Aberystwyth University, Aberystwyth, Ceredigion, SY23 3FL
[13]Laboratory of Optical Astronomy and Solar-Terrestrial Environment, Institute of Space Sciences, School of Space Science and Physics, Shandong University, Weihai, Shandong, China.

[14]Harvard-Smithsonian Center for Astrophysics, Smithsonian Astrophysical Observatory, Cambridge, MA, USA

[15]Department of Physics and Astronomy, University of Leicester, Leicester, LE1 7RH, UK

[16]Department of Physics, Lancaster University, Lancaster, UK

Contents of this file

Text S1
Figures S1
Tables S1

Text S1.

To explore the difference between a uniformly distributed probability of X-ray emission from region X (i.e. each latitude-longitude location in region X has an equal probability of being the source of the simulated X-ray) and the UV-driven X-ray probability distribution outlined in the main text, we ran 100,000 simulations with a uniform distribution for each observation. An example simulation run is shown in Figure S1, with the resulting counts for all observations in Table S1. For all observations the mean from the UV distribution is within 1 standard deviation of the mean for a uniform distribution.

Alongside this, Table S1 provides the number of observed counts per square degree within region X and the DPR. Then, using this value, the table provides an expected counts in region X if it produced the same number of photons per square degree as the DPR ('if the DPR was region X-like'). Similarly, the table also provides an expected number of counts in the DPR if it produced the same number of counts per square degree as Region X ('if Region X was DPR-like'). This accounts for the difference in spatial size of each region from observation to observation and highlights the discrepancy in X-rays detected in each of the two regions.

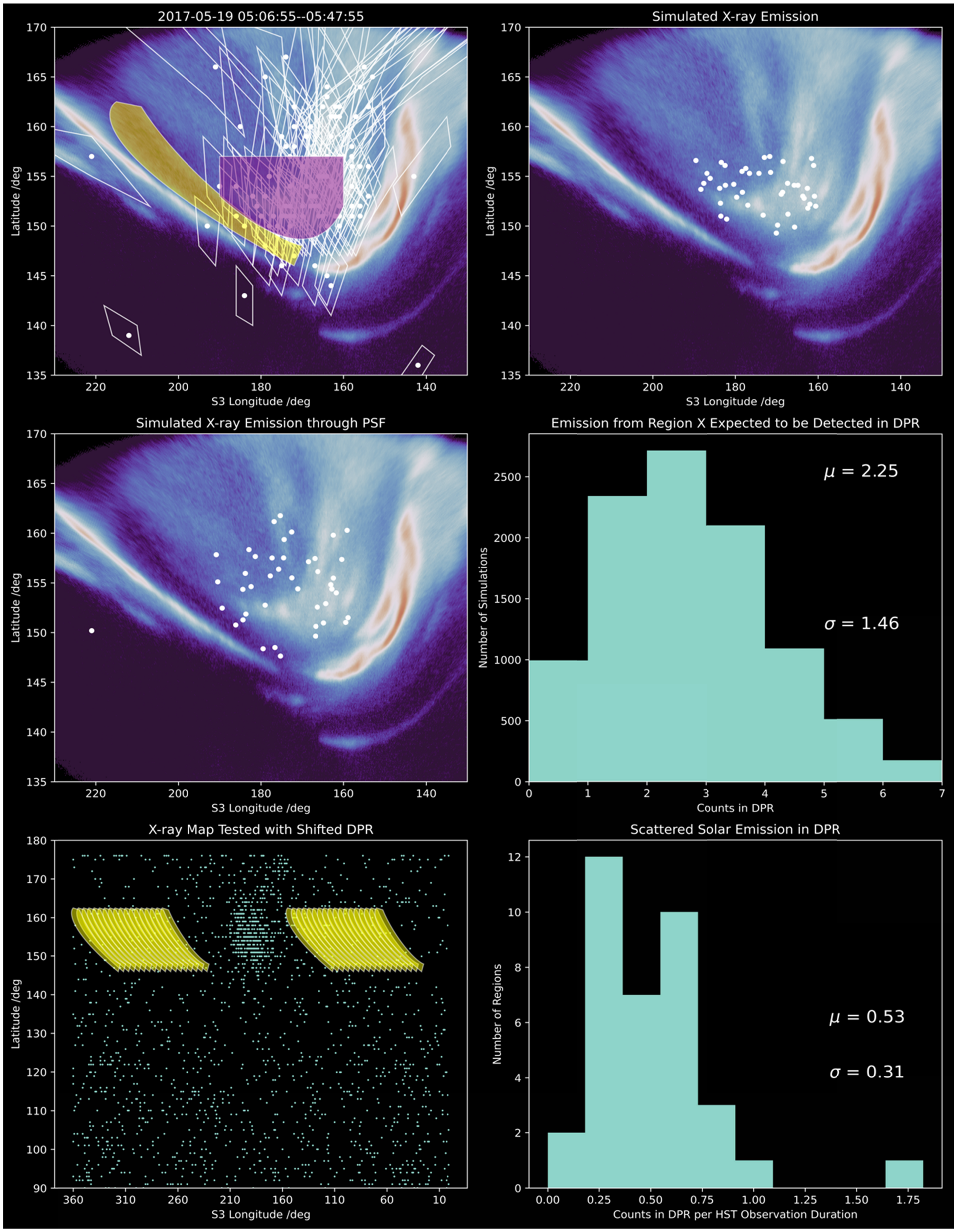

Figure S1. *Same as Figure 2 in the main text, but using a uniform probability to determine the longitude-latitude location from which an X-ray is generated in region X (purple region in the top left panel), rather than deriving the probability distribution from the UV brightness in region X.*

CXO-HST Joint Observation<br><br>YYYY-MM-DD T hh:mm-hh:mm	DPR Counts	Region X Counts	DPR Photons / sq deg	Region X Photons / sq deg	Expected DPR counts if region X-like	Expected region X counts if DPR-like	Expected Region X X-rays Detected in DPR if Uniformly Distributed
2016-05-24 T17:45-18:29	0	26	0	0.1	13	0	1.6±1.2
2016-05-24 T20:56-21:40	3	23	0.03	0.1	16	4	0.7±0.8
2016-06-01 T14:57-15:41	4	12	0.009	0..04	18	3	1.4±0.4
2016-06-01 T18:08-18:52	3	9	0.01	0.02	6	5	0.6±0.8
2017-02-02 T16:58-17:38	1	11	0.01	0.04	3	4	0.4±0.6
2017-03-27 T08:41-09:21	0	7	0	0.02	2	0	0.1±0.3
2017-05-19 T05:06-05:46	4	39	0.03	0.2	29	5	2.3±1.4
2017-05-19 T06:42-07:22	0	24	0	0.08	9	0	1±1
2018-04-01 T10:38-10:56	0	12	0	0.06	9	0	0.7±0.8
2018-05-24 T09:39-10:09	1	15	0.004	0.05	15	1	0.7±0.8
2018-09-07 T05:10-05:50	1	1	0.007	0.004	1	2	0.1±0.3

2019-07-15 T14:43-15:21	3	17	0.01	0.06	13	4	2.1±1.4
2019-07-15 T16:16-16:54	3	16	0.02	0.07	10	5	0.8±0.9
2019-07-16 T11:20-11:58	3	24	0.02	0.11	16	4	2±1

Table S1. *X-ray counts from the DPR for the time intervals shown in the first column. Northern aurora X-ray photons are only included from the time window in the first column. The columns compare the number of X-ray photons detected in the DPR and region X, the number of photons per square degree in the DPR and region X. From these values, we calculated the expected number of detected photons in the DPR if it was region X like and in region X if it was DPR-like. This highlights the clear difference in photon distribution between the two regions. The final column shows the number of photons from the active and swirl region that are expected to be detected in the DPR because of uncertainties in the photon spatial location (see e.g. Figure 3C and D) as mean±standard deviation from 100,000 simulation runs, based on a simulated uniform probability across region X.*
